# Tryptophan Hydroxylase 1 Regulates Tryptophan and Its Metabolites

**DOI:** 10.3390/ijms26093978

**Published:** 2025-04-23

**Authors:** Katsunori Nonogaki, Takao Kaji

**Affiliations:** Division of Diabetes and Nutrition, RARiS, Tohoku University, 6-6-11 Aramakiaza-Aoba, Aoba-ku, Sendai 980-8579, Miyagi, Japan

**Keywords:** Tph1, serotonin, tryptophan, indolepropionic acid, kynurenine, brain, FGF21, blood glucose, food intake

## Abstract

Tryptophan hydroxylase (Tph), the rate-limiting enzyme of serotonin (5-hydroxytryptophan; 5-HT) synthesis, exists in two isoforms, Tph1 and Tph2. Tph1 upregulates peripheral 5-HT synthesis and blood 5-HT levels, whereas Tph2 upregulates brain 5-HT synthesis. Here, we show that plasma and brain levels of tryptophan and its metabolites, including 5-HT, 5-hydroxy indoleacetic acid, indolepropionic acid, indole-3-acetic acid, kynurenine, and xanthurenic acid, are decreased in young (8-week-old) Tph1-mutant mice compared with age-matched wild-type mice. In older (7-month-old) Tph1-mutants, the decreases in tryptophan and its metabolites outside the 5-HT pathway were diminished. Although single-housed Tph1 mutants displayed age-related alterations in food intake, body weight, and plasma FGF21 levels, blood glucose levels were lower in both young and older Tph1 mutants compared with age-matched wild-type mice. These findings suggest that Tph1 regulates tryptophan and its metabolites in the plasma and brain, as well as blood glucose homeostasis.

## 1. Introduction

Tryptophan hydroxylase (Tph) is the rate-limiting enzyme in the biosynthesis of serotonin. Tph catalyzes the biopterin-dependent monooxygenation of tryptophan to 5-hydroxytryptophan, which is subsequently decarboxylated by aromatic amino acid decarboxylase to form serotonin (5-hydroxytryptamine; 5-HT).

Since the discovery of the two isotypes of Tph (Tph1 and Tph2) [1], it has become widely accepted that the central and peripheral 5-HT systems are functionally separate. The spatial separation is attributable to the inability of 5-HT to cross the blood–brain barrier (BBB); thus, 5-HT is biosynthesized centrally by Tph2 and peripherally by Tph1 [1,2,3,4].

Tph1 is mainly expressed in the gut and in other peripheral tissues and supplies platelets in the circulation with 5-HT [1,2,3,4]. Studies using Tph1 mutant mice revealed that gut-derived 5-HT has hormonal effects on liver, bone, heart, and blood vessels, whereas locally synthesized 5-HT in the peripheral tissues including pancreas, bone marrow, bone, gut, mammary gland, has paracrine and/or autocrine effects [3]. Genetic ablation of Tph1 reportedly stops β-cell proliferation in the pancreas, leading to impaired glucose tolerance [5].

On the other hand, recent studies revealed that genetic ablation of Tph1 improves glucose tolerance in mice fed chow diet [6], and gut microbiome can mediate the gut-derived 5-HT [6]. Moreover, genetic ablation of Tph1 can protect against high-fat diet-induced obesity and hepatic steatosis in mice [7,8]. It is therefore suggested that peripheral 5-HT promotes obesity and hepatic steatosis by increasing insulin secretion and de novo lipogenesis in white adipose tissue and liver, and inhibiting the thermogenesis in beige and brown adipose tissue [9].

High expression of Tph1 was recently reported in the human pituitary [4], and polymorphisms of Tph1 may be associated with an elevated risk for obesity [10], suicide behavior [11] and schizophrenia [12] in humans. The roles of Tph1 in brain 5-HT synthesis as well as in tryptophan and its metabolites, however, remain unclear.

In the present study, we demonstrated alterations in tryptophan and its metabolites in the plasma and brain of young (8-week-old) and older (7-month-old) Tph1 mutant mice compared with age-matched wild-type mice. We also revealed age-related alterations in body weight, food intake, and blood glucose levels in single-housed Tph1 mutants.

## 2. Results

### 2.1. Plasma Tryptophan and Its Metabolites in Tph1 Mutants and Wild-Type Mice

Plasma 5-HT levels were remarkably decreased in Tph1 mutants to 3% of the levels in age-matched wild-type mice (Table 1a). Significant decreases were also observed in the plasma levels of l-tryptophan (Trp), 5-hydroxy indoleacetic acid (5-HIAA), kynurenine (KYN), xanthurenic acid (XA), indole-3-propionic acid (IPA), indole-3-acetic acid (IAA), and kynurenic acid (KYNA) levels in 8-week-old Tph1 mutant mice compared with age-matched wild-type mice (Table 1a). These findings suggest that Tph1 upregulates not only peripheral 5-HT synthesis but also Trp and its metabolites involved in the KYN and IPA pathways in 8-week-old mice.

Plasma levels of 5-HT and 5-HIAA were significantly decreased in 7-month-old Tph1 mutants compared with age-matched wild-type mice, whereas no significant differences were detected in the plasma levels of Trp, KYN, XA, and IPA between 7-month-old Tph1 mutants and age-matched wild-type mice (Table 1a). Rather, plasma IAA levels were significantly increased in 7-month-old Tph1 mutants compared with age-matched wild-type mice (Table 1a). Thus, Tph1 mutants exhibited age-related changes in the plasma levels of Trp and its metabolites involved in the KYN and IPA pathways.

### 2.2. Brain Tryptophan and Its Metabolites in Tph1 Mutants and Wild-Type Mice

In the brain, Trp, 5-HT, 5-HIAA, XA, IPA, and IAA levels were significantly decreased in 8-week-old Tph1 mutants compared with age-matched wild-type mice (Table 1b). Brain levels of Trp, 5-HT, 5-HIAA, KYN, and IPA were significantly decreased in 7-month-old Tph1 mutants compared with age-matched wild-type mice (Table 1b), whereas there were no significant differences in brain levels of XA and IAA (Table 1b). These results suggest that Tph1 is involved in the upregulation of brain Trp, 5-HT, KYN, and IPA, but the upregulation is likely to be smaller in older mice than in young mice.

### 2.3. Body Weight, Food Intake, Blood Glucose and Plasma FGF21 and Insulin Levels in Tph1 Mutants and Wild Type Mice

**Figure 1:**
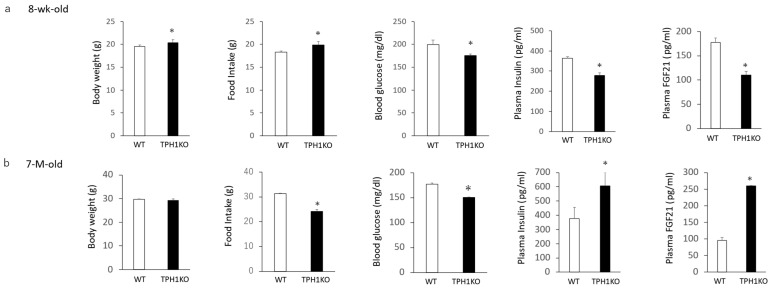
Body weight changes, body weight, food intake for 6 days, blood glucose levels, plasma insulin, and plasma FGF21 levels in single-housed 8-week-old (**a**) and 7-month-old (**b**) Tph1 mutants and wild-type mice. Data are presented as the mean ± SEM (n = 6/group). * *p* < 0.05 from wild-type.

Body weight and total food intake for 6 days after the singly housed condition were significantly increased in single-housed 8-week-old Tph1 mutants compared with age-matched wild-type mice, whereas blood glucose, plasma FGF21, and plasma insulin levels were significantly decreased (Figure 1a). On the other hand, age-related differences were observed in older mice. There were no significant differences in body weights between 7-month-old Tph1 mutants and wild-type mice. Total food intake for 6 days after the singly housed condition, and blood glucose levels were significantly decreased in single-housed 7-month-old Tph1 mutants compared with age-matched wild-type mice, and plasma FGF21 and insulin levels were significantly increased (Figure 1b). Thus, despite the age-related alterations in body weight, food intake, and plasma FGF21 and insulin levels in single-housed Tph1 mutants, blood glucose levels were lower in both young and older Tph1 mutants compared with wild-type mice.

## 3. Discussion

The results of the present study revealed that Tph1 is required for maintaining not only the plasma levels of 5-HT but also the plasma levels of Trp and its metabolites, including KYN, XA, IPA, and IAA, in young mice. Although Trp is an essential amino acid that cannot be synthesized in the body and must be obtained from the diet, our results revealed that Tph1 partially regulates Trp levels in the plasma and brain. Circulating Trp can enter the brain through the BBB, and thus the decreased brain Trp levels in Tph1 mutants may result from a decrease in the plasma Trp levels. The decreased plasma and brain metabolite levels in the KYN and IPA pathways in Tph1 mutants may result from the decrease in Trp.

In addition, our results revealed that Tph1 is involved in brain 5-HT synthesis in young and older mice. Peripheral 5-HT cannot cross the BBB, and Tph1 mRNA and protein are not detected in the mouse brain [2]. Brain 5-HT synthesis is maintained by Tph2 in mice [1,2,3,4]. Accordingly, brain 5-HT synthesis induced by Tph2 might be not maintained in Tph1 mutants. The decreases in plasma and brain Trp levels might also contribute to the decrease in brain 5-HT in Tph1 mutants.

Moreover, our results revealed that aging can affect Trp and its metabolites in the plasma and brain. Plasma Trp and its metabolites outside the 5-HT pathway tended to be lower in older wild-type mice than in young wild-type mice, but these age-related differences were attenuated in Tph1 mutants. Rather, metabolites in the plasma IPA pathway tended to increase in older Tph1 mutants compared with the age-matched wild-type mice. The increases in plasma Trp and Trp-derived metabolites in older Tph1 mutants might result from the compensatory mechanisms in the absence of Tph1.

Similarly, brain Trp and its metabolites tended to be lower in older wild-type mice than young wild-type mice; the differences in Trp and its metabolites between wild-type mice and Tph1 mutants were smaller in older mice than young mice. The decreases in brain Trp and its metabolites in older wild-type mice might result from the decreases in plasma Trp in older wild-type mice. We also cannot rule that the decrease in brain Tph2 function might be involved in the decreases in brain Trp and its metabolites in older wild-type mice.

Our results support our previous findings that food intake and body weight are increased in association with decreases in hepatic FGF21 expression and plasma FGF21 levels in young Tph1 mutants [13], suggesting that 5-HT upregulates hepatic FGF21 production. The present results, however, also revealed that food intake and body weight are decreased in association with increases in plasma levels of FGF21 and insulin in single-housed older Tph1 mutants. Because Trp may affect food intake [14], the age-related alterations of Trp levels in plasma and brain might be involved in the age-related alterations of food intake in Tph1 mutants. In addition, the age-related alterations of plasma FGF21 levels might be related to the age-related alterations in food intake and body weight. Moreover, the other neural transmission in the brain might contribute to the age-related alterations of food intake and body weight in Tph1 mutants.

The age-related alterations in plasma FGF21 might be related to age-related changes in plasma IAA levels. FGF21 is implicated in the regulation of glucose uptake and metabolism [15,16,17]. Despite the age-related alterations in food intake, body weight, and plasma levels of FGF21 and insulin, blood glucose levels were lower in both young and older Tph1 mutants than in age-matched wild-type mice. These results suggest that 5-HT synthesis via Tph1 contributes to blood glucose homeostasis independently of feeding, body weight, insulin, and FGF21.

Mechanisms by which aging can alter Trp and several Trp-derived metabolites in plasma and brain, food intake, body weight, plasma FGF21 and insulin levels in Tph1 mutants and wild-type mice, and mechanisms by which Tph1 can regulate blood glucose levels, remain unclear. Further studies will need to determine them in the future.

In summary, these findings suggest that Tph1 is required for the regulation of Trp and its metabolites involved in the KYN and IPA pathways, in plasma and brain, and that Tph1 is required for blood glucose homeostasis.

## 4. Materials and Methods

### 4.1. Tph1 Mutant Mice

Homozygous mutant males bearing a null mutation of the Tph1 gene (congenic on a C57BL/6N background) and age-matched wild-type mice were used. The line was maintained through mating of females heterozygous for the Tph1 gene with heterozygous males obtained from Cyagen Biosciences Inc. (Santa Clara, CA, USA). Genomic DNA was extracted from tails of littermates using TaKaRa MiniBEST Universal Genomic DNA Extraction kit (Ver.5.0_Code No. 9765, Kusatsu, Japan). Genotypes were confirmed by PCR-LabChip (PerkinElmer LabChip GX Touch HT, Yokohama, Japan) analysis using the forward primer F1: 5′-ACATCAGCCTTCTGCTCTGTTTC-3′ and the reverse primer R1: 5′-TCACTGAGAGCATCAAGCCCAG-3′ and R2: 5′-ATTTCCGGGACTCGATGTGTAAC-3′. Tph1 mutant and wild-type alleles correspond to the 611- and 489-bp fragments, respectively.

In the wild-type allele, the forward primer F1 and the reverse primer R1 span 14,449 bp of the Tph1 gene on mouse chromosome 7, prior to the deletion of exons in the Tph1 gene. Due to the deletion of exons in the Tph1 gene in the mutant allele, the distance between the forward primer F1 and the reverse primer R1 is 611 bp. Moreover, we confirmed homozygous Tph1 mutants by determining plasma 5-HT levels using a mouse serotonin ELISA kit as described previously [13,18].

Animals were all housed (3–5 mice per cage) with free access to water and chow pellets in a light- (12 h on/12 h off; lights off at 2000 h) and temperature (20–22 °C)-controlled environment. The animals were singly housed in cages for 1 week before the experiment. Body weights were measured weekly for 9 weeks, starting at 8 weeks of age. The final body weights and food intake for 6 days were measured after the singly housed conditions. The animal studies were conducted in accordance with the institutional guidelines for animal experiments at Tohoku University Graduate School of Medicine and all experimental protocols were approved by the institutional committee at Tohoku University.

### 4.2. Blood Chemistry

Whole blood was mixed with EDTA-2Na (2 mg/mL) and aprotinin (500 kIU/mL) to determine the plasma levels of FGF21 and insulin. Plasma levels of FGF21 and insulin were measured by enzyme-linked immunosorbent assay (rat/mouse FGF21 ELISA Kit, R&D Systems, Tokyo, Japan, and a mouse Insulin ELISA Kit [TMB], AKRIN-011T, Shibayagi, Gunma, Japan, respectively) as described previously [13,18]. Blood glucose levels were measured using glucose strips (blood glucose monitoring system; Accu-Check, Roche Diagnostics, Tokyo, Japan).

### 4.3. Tryptophan and Its Metabolites Analysis

Tryptophan and its metabolites were subjected to analysis by LSI Medience Corporation, a contracted laboratory based in Tokyo, Japan as described previously [18]. Briefly, brain and plasma were introduced into sample disruptor tubes provided by Yasui Kikai (Osaka, Japan). Subsequently, these tubes were agitated with iron cones that had been pre-cooled in liquid nitrogen. The resulting sample powders were suspended in 500 µL of methanol and vigorously shaken for 15 min, after which centrifugation was carried out at 20,000× *g* for 3 min. The supernatants, constituting 40 µL, were meticulously transferred to 2 mL microtubes. Internal standards were then introduced and combined with the supernatants, followed by the addition of 1000 µL of a 2% formic acid solution to induce protein precipitation. Afterwards, we purified the analytes from the supernatant using solid-phase extraction (OASIS MCX, Waters, Milford, MA, USA) and analyzed them using liquid chromatography-tandem mass spectrometry (Ultivo, Agilent, Santa Clara, CA, USA) with a reverse-phase LC column (ACQUITY UPLC HSS T3, 1.8 µm, 2.1 mm × 50 mm, Waters, Milford, MA, USA). The data were processed with Mass Hunter software (Agilent, Santa Clara, CA, USA). We normalized the peak areas using internal standards and determined the concentration of each analyte using a standard curve.

### 4.4. Statistical Methods

Data are presented as mean ± SEM (n = 6). Comparisons between the two groups were performed using Student’s *t*-test. A *p* value of less than 0.05 was considered statistically significant.

## Figures and Tables

**Table 1:** (**a**) Plasma Trp and its metabolites in Tph1 mutants and wild-type mice; (**b**) Brain Trp and its metabolites in Tph1 mutants and wild-type mice.

(**a**)			
**Plasma**	**8-Week-Old** **WT**	**8-Week-Old** **TPH1KO**	**% of WT**
Trp	21,133.3 ± 2168.9	12,025 ± 663.79 *	57%
5-HT	256.66 ± 33.2	6.42 ± 0.46 *	3%
5-HIAA	29.93 ± 1.22	15.45 ± 1.9 *	52%
KYN	115.36 ± 14.54	88.55 ± 7.47 *	77%
XA	155.4 ± 12.7	90.9 ± 4.2 *	58%
IPA	853.5 ± 35.5	665.75 ± 43.48 *	78%
IAA	160.33 ± 16.65	93.07 ± 17.66 *	58%
KYNA	9.13 ± 0.95	5.1 ± 0.39 *	56%
**Plasma**	**7-Month-Old** **WT**	**7-Month-Old** **TPH1KO**	**% of WT**
Trp	18,100 ± 2056.1	16,400 ± 1675.7	91%
5-HT	498 ± 141.2	3.54 ± 0.625 *	1%
5-HIAA	21.3 ± 0.86	10.19 ± 0.26 *	48%
KYN	73.8 ± 2.26	76.78 ± 7.47	104%
XA	113.9 ± 11.3	121.2 ± 17.95	106%
IPA	753.3 ± 99.4	869.3 ± 114.08	115%
IAA	71.1 ± 6.58	96.66 ± 7.38 *	136%
KYNA	4.54 ± 0.65	5.53 ± 1.05	122%
(**b**)			
**Brain**	**8-Week-Old** **WT**	**8-Week-Old** **TPH1KO**	**% of WT**
Trp	3710 ± 44.8	2755 ± 117 *	74%
5-HT	444 ± 15.5	378 ± 9 *	85%
5-HIAA	370 ± 13.8	316 ± 7 *	85%
KYN	28.77 ± 2.8	24.48 ± 1.2	85%
XA	60.0 ± 1.3	46.0 ± 2.0 *	77%
IPA	7.96 ± 0.56	5.36 ± 0.07 *	67%
IAA	13 ± 0.3	11 ± 0.1 *	85%
**Brain**	**7-Month-Old** **WT**	**7-Month-Old** **TPH1KO**	**% of WT**
Trp	2613 ± 102	2530 ± 131.8 *	97%
5-HT	423 ± 13.2	375 ± 10.3 *	89%
5-HIAA	346 ± 3.4	306 ± 15.1 *	88%
KYN	15.53 ± 0.31	14.8 ± 0.04 *	95%
XA	45.0 ± 1.6	47.0 ± 2.2	104%
IPA	8.52 ± 0.42	7.20 ± 1.22 *	85%
IAA	11 ± 0.3	11 ± 0.2	100%

Trp; L-tryptophan, 5-HT; serotonin, 5-HIAA; 5-hydroxy indole-3-acetic acid, KYN; L-kynurenine, XA; xanthurenic acid, and IPA; indole-3-propionic acid. * *p* < 0.05 from WT.

## Data Availability

Some or all datasets generated during and/or analyzed during the current study are not publicly available but are available from the corresponding author on reasonable request.
